# Incidence of and outcomes of pregnancy in adolescents with perinatally-acquired HIV infection in Southern Africa

**DOI:** 10.1177/09564624251352066

**Published:** 2025-06-27

**Authors:** Nyasha Veronica Dzavakwa, Molly Chisenga, Hilda Banda-Mabuda, Cassandra Namukonda, Lackson Kasonka, Tsitsi Bandason, Nicol Redzo, Hilda Angela Mujuru, Katharina Kranzer, Victoria Simms, Rashida A Ferrand

**Affiliations:** 1 The Health Research Unit Zimbabwe, Biomedical Research and Training Institute, Harare, Zimbabwe; 2 4906 Department of Infectious Disease Epidemiology and International Health, London School of Hygiene and Tropical Medicine, London, UK; 3 University Teaching Hospitals, Women and Newborn Hospital, Lusaka, Zambia; 4 37595 Department of Child, Adolescent and Women Health, University of Zimbabwe, Harare, Zimbabwe; 5 559150 Department of Clinical Research, London School of Hygiene and Tropical Medicine, London, United Kingdom; 6 Department of Infectious Disease and Tropical Medicine, Centre for Infectious Diseases, Heidelberg University Hospital, Germany

**Keywords:** Adolescent HIV, pregnancy incidence, perinatally acquired HIV, birth outcomes, contraceptive use

## Abstract

**Background:**

The scale-up of antiretroviral therapy (ART) has enabled more children living with HIV to reach adolescence and become sexually active. We investigated pregnancy incidence and outcomes among adolescents with perinatally acquired HIV enrolled in a multi-country trial of vitamin D and calcium carbonate supplementation (VITALITY; PACTR202009897660297).

**Methods:**

Between February and November 2021, 842 adolescents aged 11–19 years from Zambia and Zimbabwe on ART for at least 6 months were enrolled. Pregnancies occurring during 96 weeks of follow-up (February 2021–October 2023) were identified through self-report or testing. Pregnancy incidence rate was calculated among post-menarche participants using survival time analysis.

**Results:**

Thirty-five adolescents (median age 18, range 13–22) became pregnant, 21 in Zambia, 14 in Zimbabwe. Overall pregnancy incidence was 4.6 per 100 person-years (95% CI 3.3–6.4), higher in those ≥15 years (6.8 per 100 person-years, 95% CI 4.8–9.7). Three pregnancies ended in miscarriage; 32 resulted in live births. Of the 30 adolescents with live births and available data, 26 (86.7%) infants were tested for HIV at birth: 24 were HIV-negative, two had unknown results. Twenty-nine received HIV prophylaxis. At 6 weeks, 19/30 infants were retested for HIV: 16 were HIV-negative. Twenty-one of 32 pregnant adolescents were in school at conception, and 4 (19.0%) returned post-pregnancy. Overall, 23/33 (69.7%) started contraception, a median of 9 weeks after delivery/miscarriage.

**Conclusion:**

Pregnancy incidence is high among adolescents with HIV, especially older adolescents. While prevention of vertical HIV transmission is effective, education re-integration and timely contraception uptake remain limited.

## Introduction

With the scale-up of antiretroviral therapy (ART), an increasing number of children living with HIV are entering adolescence and adulthood.^1^ Consequently, there is now a growing third generation of HIV-exposed individuals, as adolescents and young people with perinatally-acquired HIV become sexually active and become pregnant.^2,3^

Adolescents face multiple barriers to sexual and reproductive health services including limited access to contraception, stigma and judgemental attitudes from providers and by communities, which may be compounded in those with HIV, increasing their risk of unplanned pregnancy.^4,5^ Furthermore, adolescents living with HIV (ALWH) face unique issues related to pregnancy.^6^ They have had life-long HIV infection, with exposure to ART either in utero and/or since infancy or later childhood, with some adolescents experiencing severe HIV-associated complications in early life which all can negatively impact pregnancy outcomes.^2,7^

Studies from high income settings have reported on pregnancy outcomes in adolescents with perinatally acquired HIV, and have found positive maternal and infant outcomes.^6,8^ Data from Africa, where the majority of adolescents living with HIV live, are scarce.^9^

We describe the incidence and outcomes of pregnancy among a cohort of adolescents with perinatally-acquired HIV who were enrolled in a clinical trial (VITALITY: PACTR202009897660297) of vitamin D and calcium carbonate supplementation to improve musculoskeletal health.^10^

## Methods

This study was embedded within a multi-country individually randomised double-blind, placebo-controlled trial (VITALITY: PACTR202009897660297) investigating the impact of high-dose vitamin D and calcium carbonate supplementation for 48 weeks on bone health among adolescents living with HIV.^10^ The trial protocol has been published. Briefly, the VITALITY trial (parent study) enrolled individuals aged 11–19 years with perinatally acquired HIV attending outpatient HIV clinics at the Women and Newborn Hospital at the University Teaching Hospital, Lusaka, and at the Children’s Hospital at Sally Mugabe Central Hospital, Harare. These are the main public sector hospitals in the two cities and serve as teaching and referral hospitals. Participants had been on antiretroviral therapy (ART) for at least 6 months and were randomised 1:1 to receive weekly high dose vitamin D (20,000iu) and daily calcium carbonate (500mg) or identical placebo for 48 weeks.

Trial participants were followed for 96 weeks after enrolment, which began in February 2021 and was completed in October 2023. Participants underwent a dual energy x-ray absorptiometry (DXA) scan at enrolment, at 48 weeks and at 96 weeks. In all female participants who reported having reached menarche, a pregnancy test was performed before each DXA scan to ensure safety. At each 12-week VITALITY (parent trial) study visit, a health check included enquiries about missed menstrual periods or possible pregnancy.

All pregnancies that occurred during the VITALITY trial follow-up period, identified through either self-report or through pregnancy tests, and details about the pregnancy and outcome were recorded using a pre-designed questionnaire administered on Android tablets. This included questions about participants’ educational, social and clinical circumstances at the time of pregnancy, relationship with the father of the unborn child, maternal perinatal ART regimen and HIV viral load. Details of antenatal care and delivery, previous pregnancies, illnesses during pregnancy, pregnancy outcomes, and contraception post-delivery were documented. Data on infant characteristics (gestational age, birth weight, history of any serious illness post birth, HIV status and type of HIV infant prophylaxis received) were collected either through maternal report or when available, extracted from the maternal antenatal outpatient clinic record or the infant birth record. However, data gaps occurred due to missing or incomplete clinic records and challenges in maternal recall, especially for events that occurred several months prior to data collection.

Pregnancy incidence rate was calculated using survival time analysis. Only those female VITALITY participants who had reached menarche were included in the denominator person time. Data were censored at the end of the VITALITY follow-up or when participants left the study.

Participants who had given written informed assent or consent to participate in the VITALITY trial gave separate written informed consent to have their pregnancy data collected. Ethical approval was obtained from the Medical Research Council of Zimbabwe (Reference number: A/2626), University of Zambia Biomedical Research Ethics Committee (Reference number:116-2020) and the London School of Hygiene and Tropical Medicine Ethics Committee (Reference number: 22030).

## Results

Eight hundred and forty-two ALWH (420 from Zambia and 422 from Zimbabwe), 448 (53.2%) female, were enrolled in the trial from February to November 2021. Of the 448 female participants enrolled 307 (68.5%) had started menarche. At 96 weeks 397 (88.6%) female participants completed follow-up; the remaining 51 had either died (*n* = 2), withdrew (*n* = 29), or were lost to follow-up (*n* = 20). During the study period 35 participants, 14 in Zimbabwe and 21 in Zambia, median age 18 (range 13–22) years, fell pregnant during the study period. Overall incidence of pregnancy was 4.6 per 100 person years (95% CI 3.3–6.4), 0.8 per 100 person years (95% CI 0.2–2.9) in those aged under 15 years and 6.8 per 100 person years (95% CI 4.8–9.7) in those who were 15 years and above.

Pregnancy outcomes were as follows: 32/35 (91.4%) pregnancies resulted in live babies and three (8.6%) were first trimester miscarriages (<12 weeks gestational age). Of the 32 participants who delivered live babies, two had no further data collected. As a result, the final analysis included 33 participants with available data: 30 who had live births and three who experienced first-trimester miscarriages. For 31/33 participants this was their first pregnancy, and two participants had one previous pregnancy aged 15 and 16 years respectively.

### Maternal characteristics, antenatal care and pregnancy outcomes

The maternal characteristics of the 33 adolescents with available data are summarised in Table 1. Twenty-one out of 32 adolescents (65.6%) were still in school when they got pregnant, and four (19.0%) went back to school post-delivery. Twenty-three out of 33 adolescents (69.7%) were on a dolutegravir based regimen. Among the 17 (51.5%) who were virally suppressed when they got pregnant (viral load <60 copies/ml), 13/17 (76.5%) were on a dolutegravir regimen, and 4/17 (23.5%) were on an efavirenz or nevirapine regimen. A third of the adolescents (12/33) were not in contact with the father of the unborn child, and more than half (7/12, 58.3%) did not know the partner’s HIV status.

**Table 1.** Maternal characteristics *N* = 33.

Variable	Category	*n* (%)
Age at pregnancy (years)	Median (range)	18 (13–22)
In school when got pregnant (*N* = 32) (missing for 1)	Yes	21 (65.6)
	No	11 (34.4)
Type of school (*N* = 18) (missing for 3)	Primary	3 (16.7)
	Secondary	12 (66.7)
	Vocational training	3 (16.7)
Gone back to school post pregnancy (*N* = 21)	Yes	4 (19.0)
	No	17 (81.0)
ART regimen at pregnancy (*N* = 33)	Dolutegravir based regimen	23 (69.7)
	PI based regimen	4 (12.1)
	NNRTI based regimen	6 (18.2)
^1^Viral load at start of pregnancy (*N* = 33)	<60 copies/ml	17 (51.5)
	≥60 copies/ml	13 (39.4)
	Viral load result not known	3 (9.1)
Relationship with partner (*N* = 33)	Not in contact	12 (36.4)
	Never married or living together	8 (24.2)
	Married or living together as if married	9 (27.3)
	Engaged or waiting for lobola	4 (12.1)
Partner’s HIV status (*N* = 33)	HIV positive	5 (15.2)
	HIV negative	17 (51.5)
	HIV status unknown	11 (33.3)
Using contraception post pregnancy (*N* = 33)	Depo	13 (39.4)
	Implant	10 (30.3)
	Did not start contraception	10 (30.3)

^1^HIV viral suppression defined as a VL <60 copies/mL, based on laboratory lower limit cutoffs for viral detection across Zambia and Zimbabwe. PI - Protease inhibitor, NNRTI - Non-nucleoside reverse transcriptase inhibitor.

Of the 30 participants with data who delivered live babies, all had registered for antenatal care with 18 (60.0%) having attended 3–4 antenatal care (ANC) visits (Table 2). Five participants were admitted to hospital during their pregnancy for shortness of breath (*n* = 1), hypotension (*n* = 1) and anaemia (*n* = 2), diagnosis at admission could not be ascertained for one participant. Information on delivery was available for 28; 26 (92.9%) had a vaginal delivery, 27 (96.4%) participants delivered at a health institution, and one delivered at home but presented to health facility on the day of delivery. One participant from Zimbabwe was admitted within 6 weeks post-delivery for a rectovaginal fistula which was repaired.

**Table 2.** Antenatal care and infant outcomes.

Variable	Category	*n* (%)
*Antenatal care in 30 with live births*		
Registered for antenatal care	Yes	30 (100)
Number of antenatal care visits	1–2	2 (6.7)
	3–4	18 (60.0)
	5–7	9 (30.0)
	12	1 (3.3)
Admitted during pregnancy (*N* = 29)	Yes	5 (17.2)
	No	24 (82.8)
Type of delivery (*N* = 28)	Vaginal	26 (92.9)
	Caesarean	2 (7.1)
Place of delivery (*N* = 28)	Hospital/clinic	27 (96.4)
	Home	1 (3.6)
Admitted for complications in 6-week post-delivery (*N* = 28)	Yes	2 (7.1)
	No	26 (92.9)
Given contraception soon after delivery (*N* = 27)	Yes	8 (29.6)
	No	19 (70.4)
Type of contraception given soon after delivery (*N* = 8)	Pills for breastfeeding women	4 (50.0)
	Injection	1 (12.5)
	Implant	3 (37.5)
*Infant outcomes of the 30 live babies*		
Gestational age at birth	33–36 weeks	5 (16.7)
	37–40 weeks	21 (70.0)
	>40 weeks	4 (13.3)
Birthweight	<2500 g	3 (10.0)
	≥2500 g	27 (90.0)

Immediately after delivery, 27 participants gave information on contraception and eight (29.6%) participants had received contraception, of whom 4/8 received short term contraception. Of the three adolescents who had a miscarriage, two did not receive contraception and one did not provide information. After a median 9 weeks post-pregnancy the number of participants who received contraception increased to 23/33 (69.7%) with all 23 receiving a long-term contraceptive method.

### Infant outcomes

Five (16.7%) out of 30 infants were delivered at <37 weeks gestational age and 3 (10.0%) had a birthweight <2500g (Table 2). Information on infant feeding was varied and was dependant on the age of the baby at the time the data was collected. Of the 29 infants whose information was collected, 7 (24.1%) were still exclusively breastfeeding, and 11 (37.9%) were on complementary feeding (mixed breastmilk and replacement feeding). Of 18 infants receiving complementary feeding or solids only, 11 (61.1%) had been breastfed exclusively for at least 24 weeks. Four infants, two each in Zambia and Zimbabwe were admitted to hospital for vomiting (*n* = 1), heart problem (*n* = 1), oral candidiasis (*n* = 1) and fever (*n* = 1). All the babies made a complete recovery. One baby died of pneumonia aged 14 months (Table 3)

**Table 3.** Infant outcomes by age.

Variable	Category	Baby’s age at interview		
		<24 weeks	≥24 weeks	Total
		20	9	29
Type of feeding now (*N* = 29)	Exclusive breastfeeding	7 (35.0)	0	7 (24.1)
	Exclusive replacement feeding	0	1 (11.1)	1 (3.5)
	Mixed breast and replacement feeding	10 (50.0)	1 (11.1)	11 (37.9)
	Solids only	3 (15.0)	7 (77.8)	10 (34.5)
Weeks of exclusive breastfeeding (*N* = 18)	<24 weeks	4	3	7 (38.9)
	≥24 weeks	6	5	11 (61.1)
Baby seriously sick or admitted in hospital (*N* = 29)	Yes	2 (10.0)	2 (22.2)	4 (13.8)
	No	18 (90.0)	7 (77.8)	25 (86.2)

.

**Figure 1.**
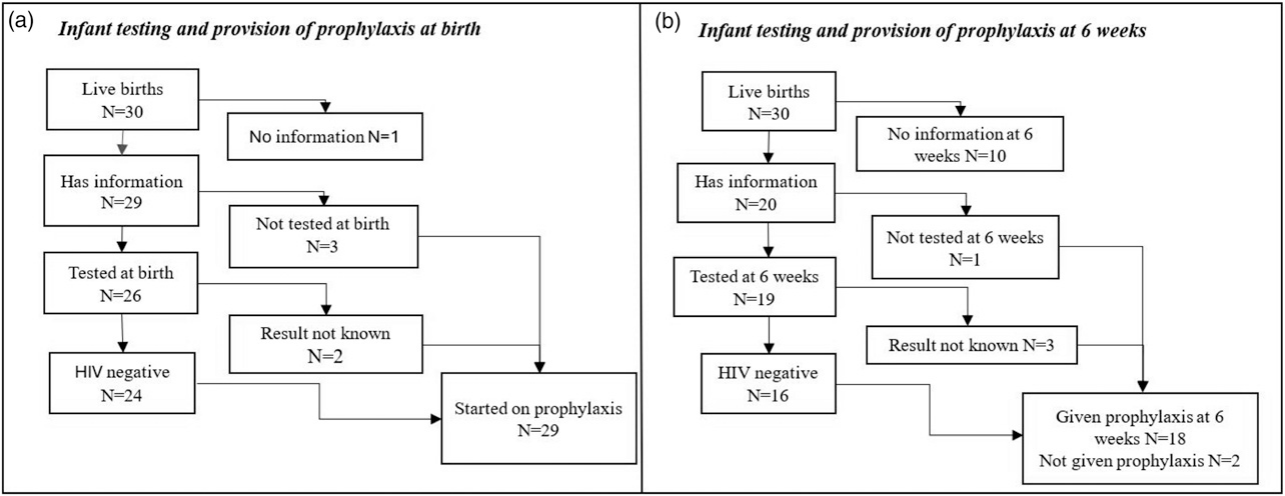
Figure 1a and 1b: Infant HIV testing and provision of prophylaxis at birth and 6 weeks.

Twenty-six infants were tested for HIV at birth and 24 (92.3%) were HIV negative, HIV test result for two infants was unknown (Figure 1(a)). All 29 infants with recorded information were started on HIV prophylaxis at birth. At 6 weeks 19/30 (63.3%) infants were re-tested for HIV, and 16/19 (84.2%) were HIV negative, for three the results were not known. Eighteen infants were continued on HIV infant prophylaxis and two were not continued. (Figure 1(b)) Reason for discontinuation of HIV infant prophylaxis was that the baby was never breastfed (*n* = 1) or unknown (*n* = 1).

## Discussion

Our study found a high incidence of pregnancy among ALWH similar to other settings in adolescents with HIV and particularly higher in those aged 15 years and above.^11^ There may have been pregnancies in adolescents who were lost to follow-up from VITALITY, so we may have underestimated the true pregnancy rate. Notably, pregnancy might be a reason for a participant to move away, be lost to follow-up or withdraw.^12^ As shown in our study birth rate rises substantially with age across adolescence.^13^ In 2023, the adolescent birth rate for girls 10–14 years in Africa was 4.4 births per 1000 women while that of girls 15–19 years was 97.9 births per 1000 women.^14^ Several factors contribute to high adolescent pregnancy rates in Africa, and these include high unmet contraception needs, gender inequalities, high rates of sexual and gender-based violence, societal pressures to bear children to establish financial security due to poor living circumstances and difficulty negotiating for safe sex practices especially in the context of non-disclosure of HIV status.^15,16^

Most girls in this study were still in school when they fell pregnant, and many did not return to school post-pregnancy. Many adolescents who fall pregnant are chased away from home and this leaves the adolescent socially and economically vulnerable, without support and the mounting responsibilities may result in school dropouts.^17,18^ Moreso, discrimination from peers, teachers and punitive measures by some schools against pregnant adolescents make it extremely challenging for adolescents to continue with education.^17^ ALWH are more likely to be orphaned and therefore may be even less likely to access support from their parents.^19^ Addressing barriers to continued education among pregnant and adolescent mothers requires multi-sectoral interventions that go beyond the education sector.^20^

Most ALWH delivered babies who were HIV uninfected despite only half being virally suppressed. This may be because the babies were delivered in health facilities where HIV infant prophylaxis was promptly initiated. Active screening for pregnancy in this trial was coupled with clear referral pathways to ANC services and follow-up to ensure early-infant diagnosis^10^ resulting in high uptake of ANC services among pregnant ALWH. However, ANC uptake may be much lower under routine conditions.^21^ Thus, most babies born from our cohort were tested for HIV at birth and received HIV infant prophylaxis soon after a birth. However, there are persisting implementation gaps as not all the babies in this cohort received birth HIV testing and for some, results were either not returned or not documented in clinic records. Three adolescents experienced an early trimester pregnancy loss. The risk of adverse pregnancy outcomes is still high in those with young age, and this may be compounded by long-standing HIV infection.^22,23^

A substantial proportion of adolescents in this study were not in stable relationships and did not know their partner’s HIV status. Challenges with disclosure of one’s HIV status to a partner is a recognised problem and can result in difficulties in negotiating for safe sexual practices.^24^ Adolescent girls with HIV may fail to interrogate their partner’s HIV status to avoid disclosing their own HIV status because of fear of rejection and the need for sustained sexual and romantic intimacy.^24^ Non-disclosure of HIV status coupled with engaging in unprotected sexual intercourse also increases the risk of HIV transmission.^25^

Contraception uptake was suboptimal post-pregnancy, given the crucial need to delay pregnancies to older age, and the high risk of adverse outcomes. Some adolescents only received short term contraception in the form of tablets soon after delivery which may not be optimal due to a high risk of non-adherence in this age-group.^26,27^ Worryingly, the reason given by the adolescents for low contraceptive uptake was lack of knowledge of where to access the services further increasing the risk of future pregnancies. This could be because most adolescents in our cohort were still receiving care from a paediatric HIV clinic where there is no provision of integrated family planning services. This underscores the critical need for establishment of models of care that proactively address the sexual and reproductive health needs of individuals as they enter adolescence.^27^ This will need to be coupled with provider training to offer respectful, non-judgemental and confidential services.^27^

## Conclusion

In summary, we report a high incidence of pregnancy among adolescents with perinatally-acquired HIV. While prevention of mother to child transmission of HIV is clearly successful, there are persisting implementation gaps that still need to be addressed. Pregnant adolescents require holistic care, monitoring and social support during and after pregnancy to ensure successful birth and maternal outcomes. As the epidemic ages and growing numbers of children with HIV enter adolescence, the need for acknowledging their sexuality and providing integrated sexual and reproductive health services is ever more pressing to ensure optimal health and quality of life.

## Data Availability

The data that support the findings of this study are available on reasonable request from the corresponding author. The data are not publicly available due to privacy or ethical restrictions.*
